# Are the KIR3DS1homozygous and KIR3DL1*h/*y+HLA-B*57 genotypes associated protection from HIV by different routes of exposure?

**DOI:** 10.1186/1742-4690-9-S2-P186

**Published:** 2012-09-13

**Authors:** B Tallon, J Bruneau, CM Tsoukas, J Routy, NF Bernard

**Affiliations:** 1 Centre de Recherche du Centre Hospitalier de l'Université de Montréal, Canada; 2 Research Institute McGill University Health Center, Montreal, Canada

## Background

We previously reported that HIV Exposed Seronegative (HESN) individuals have a higher frequency than HIV infected subjects of 2 genotypes: homozygosity for Killer Immunoglobulin-like Receptor (KIR) 3DS1 (KIR3DS1hmz) and homozygosity for high expression KIR3DL1 genotypes co-expressed with HLA-B*57 (*h/*y+B*57). KIR3DL1/S1 are Natural Killer (NK) cell receptors that influence NK functionality. Here, we assessed whether these genotypes were associated with protection by parenteral and mucosal routes of HIV exposure.

## Methods

473 Caucasian individuals were typed for HLA, KIR3DL1/S1 generic genotypes and KIR3DL1 allotypes. Of 88 HESN, n=69 were injection drug users (IDU) and 19 were sexually exposed (sHESN). Of 385 HIV seropositive subjects n=108 were IDU and n=277 were infected through sexual exposure. The frequency of KIR3DS1hmz and *h/*y+B*57 carriers was compared in HESN versus HIV susceptible subjects exposed through parenteral versus mucosal routes.

## Results

KIR3DS1hmz were more frequent among HESN than HIV positive IDU (10% versus 2.7%, respectively, p=0.05). This genotype was also more frequent among HESN than HIV infected individuals exposed sexually (25% versus 5.7%, respectively, p<0.01). The *h/*y+B*57 genotype was more frequent among HESN than HIV positive IDU (7.2 vs. 0%, respectively, p<0.01). This genotype was not observed among any sHESN and was detected in 1.8% of mucosally HIV infected individuals (p=not significant).

## Conclusion

The protective HESN KIR3DS1hmz genotype is associated with protection from HIV infection by both mucosal and parenteral routes. *h/*y+B*57 carriers are more frequent among IDU HESN than HIV susceptible subjects suggesting a protective effect via exposure by this route. Although there is no evidence that the *h/*y+B*57 genotype is protective at the level of sexual exposure the small number of sHESN precludes making firm conclusions on this point. Carriage of both these genotypes is linked to potency of NK cell function, which may influence early innate responses to HIV.

